# Enhanced Synthesis of Volatile Compounds by UV-B Irradiation in *Artemisia argyi* Leaves

**DOI:** 10.3390/metabo14120700

**Published:** 2024-12-11

**Authors:** Haike Gu, Zhuangju Peng, Xiuwen Kuang, Li Hou, Xinyuan Peng, Meifang Song, Junfeng Liu

**Affiliations:** 1 Institute of Radiation Technology, Beijing Academy of Science and Technology, Beijing 100875, China; 2 College of Life Science and Technology, Beijing University of Chemical Technology, Beijing 100029, China; 3 School of International Education, Beijing University of Chemical Technology, Beijing 100029, China; 4 National Natural History Museum of China, Beijing 100050, China

**Keywords:** volatile compound, *Artemisia argyi*, UV-B radiation, transcription factor

## Abstract

**Background:** Volatile compounds have a deep influence on the quality and application of the medicinal herb *Artemisia argyi*; however, little is known about the effect of UV-B radiation on volatile metabolites. **Methods:** We herein investigated the effects of UV-B exposure on the volatile compounds and transcriptome of *A. argyi* to assess the potential for improving its quality and medicinal characteristics. **Results:** Out of 733 volatiles obtained, a total of 133 differentially expressed metabolites (DEMs) were identified by metabolome analysis. These were classified into 16 categories, primarily consisting of terpenoids, esters, heterocyclic compounds, alcohols, and ketones. Sensory odor analysis indicated that green was the odor with the highest number of annotations. Among the 544 differentially expressed genes (DEGs) identified by transcriptome analysis, most DEGs were linked to “metabolic pathways” and “biosynthesis of secondary metabolites”. Integrated analysis revealed that volatiles were mainly synthesized through the shikimate pathway and the MEP pathway. RNA-seq and qPCR results indicated that transcription factors HY5, bHLH25, bHLH18, bHLH148, MYB114, MYB12, and MYB111 were upregulated significantly after UV-B radiation, and were therefore considered key regulatory factors for volatiles synthesis under UV-B radiation. **Conclusions:** These findings not only provide new insights into UV-induced changes in volatile compounds, but also provide an exciting opportunity to enhance medicinal herbs’ value, facilitating the development of products with higher levels of essential oils, flavor, and bioactivity.

## 1. Introduction

*Artemisia argyi* is a herbaceous medicinal plant that is widely distributed and used in China and other parts of the world [1,2]. The commercial value of medicinal plants mainly depends upon the production of secondary metabolites, including phenolics, terpenoids, alkaloids, tannins, lignans, and organic acids [3,4]. The peculiar aroma produced by *A. argyi* can not only prevent and treat diseases, but also purify air and repel mosquitoes [2,4]. Since the volatile aromatic oils are responsible for plant-to-plant signaling, plant thermotolerance, pathogen protection, attracting herbivores’ natural enemies, etc., they are also regarded as plant’s immune system [5,6,7,8]. The content of volatile oils varies significantly depending on species, environmental conditions, and even extraction conditions [9]. Perhaps not surprisingly, the yield and components of plant volatile oils are tightly regulated by multiple abiotic factors, such as water, temperature, light intensity, and spectral composition [10].

In the last decades, the elevated UV-B radiation caused by ozone depletion has raised concerns about the impact of UV-B on ecosystems. Though UV-B (280–315 nm) is only a small part of the solar spectrum, it not only has a significant effect on plant growth and development, but also alters the composition of metabolites in plants and the nutritional value of crops for human diet [11,12,13]. Enhanced UV-B radiation negatively affects plant physiological processes, growth, and productivity [14]. A common response to UV-B radiation is oxidative stress, with elevated levels of reactive oxygen species [15]. To reduce the oxidative stress, the comprehensive regulation of molecular and physiological responses includes the accumulation of protective UV-absorbing compounds, induction of antioxidant mechanisms, and more compact aboveground plant structure [16,17,18]. However, some medicinal plants positively respond to UV-B by increasing secondary metabolite production [4,19]. So, UV-B radiation may be an important tool for enhancing the health beneficial properties of medicinal plants [20].

Changes in volatile compounds caused by UV-B irradiation have been described in some fruits and vegetables. For example, UV-B can induce grape berries and grape skins to produce more volatile compounds (mainly monoterpenes) and affect wine flavor [21,22]. Compared with the well-watered treatment without UV-B, the volatile compounds in *Vitis vinifera* L. cv. Pinot Noir are slightly increased under UV-B radiation and water deficit conditions [23]. On the contrary, the total volatile content in leaves of *Flourensia cernua* is higher under UV-restricted treatment than the control [24]. UV Resistance Locus 8 (UVR8) is the only known plant photoreceptor that mediates the light response to UV-B [25]. UVR8 senses the UV-B signal through photo-induced dimer dissociation, which triggers a wide range of cellular responses related to photomorphogenesis and photoprotection. Currently, the regulation of the UVR8-mediated signal transduction module on the synthesis of volatile compounds under UV-B irradiation still remains obscure.

In our previous work, the critical role of flavonoids in the adaptation of *A. argyi* to UV radiation has been investigated [4]. However, knowledge about the effect of UV-B radiation on the volatile metabolites of *A. argyi* is scare, which to some extent restricts the application of the volatile essential oil of *A. argyi*. The aim of this work was to investigate the effect of UV-B radiation on the profile of volatile compounds, and its possible molecular regulation. The integrated metabolomics and transcriptomics analysis was conducted to characterize key volatile components and genes. This work provided valuable guidance for the application of UV-B radiation in medicinal plants’ production.

## 2. Materials and Methods

### 2.1. Plant Materials

The *A. argyi* cultivar NYYY was obtained from Guoyizhongjing Wormwood Industry Co., Ltd. (Nanyang, China). NYYY was planted in soil at 25 °C for 2 months under a light/dark cycle of 16/8 h, with a light intensity of 100 μmol/m^2^/s, and then transferred to 24 h light for 10 days. Afterwards, the plants were treated with UV-B (280–315 nm) radiation (2.5 μmol/m^2^/s) and the leaves were collected at 0 h, 4 h, 8 h, and 6 days, respectively. These leaf samples were immediately frozen in liquid nitrogen and stored at −80 °C for further analysis.

### 2.2. Metabolite Extraction and GC–MS Analysis

A total of 12 leaf samples harvested at 0 h and 6 days were subjected to liquid nitrogen grinding and vortex mixing, with 500 mg of each sample taken and placed in a headspace bottle. Then, saturated NaCl solution and 20 µL of 10 µg/mL internal standard (Sigma-Aldrich, Saint Louis, MO, USA) solution were added separately, and headspace solid-phase microextraction (HS-SPME) was employed to extract volatile metabolites. After the mixture was oscillated at 60 °C for 5 min, the extraction head 120 µm DVB/CWR/PDMS (Agilent, Santa Clara, CA, USA) was inserted into the sample headspace bottle. The extraction head was aged at 250 °C for 5 min in the Fiber Conditioning Station (CTC Analytics AG, Zwingen, Switzerland) before sampling. Headspace extraction was performed for 15 min, and desorbed at 250 °C for 5 min.

GC–MS (Agilent, Santa Clara, CA, USA) was performed to separate and identify volatile metabolites. The GC was equipped with a 30 m × 0.25 mm × 0.25 μm DB-5MS capillary column (Agilent, Santa Clara, CA, USA), which used helium (purity not less than 99.999%) as carrier gas with a linear velocity of 1.2 mL/min. The injector temperature was 250 °C, the oven temperature was maintained at 40 °C for 3.5 min, increased to 100 °C at 10 °C/min, then increased to 180 °C at 7 °C/min, and finally increased to 280 °C at 25 °C/min and maintained for 5 min. Mass spectrometry was carried out as follows: electron bombardment ion source (EI), ion source temperature 230 °C, quadrupole temperature 150 °C, interface temperature 280 °C, electron energy 70 eV, selected ion detection mode (SIM), and qualitative and quantitative ion precise scanning (GB 23200.8-2016). The qualitative and quantitative analysis of metabolites were completed by Metware Biotechnology Co., Ltd. (Wuhan, China). Differentially expressed metabolites (DEMs) were identified with thresholds of |log_2_Fold Change| ≥ 1 and FDR < 0.05.

### 2.3. RNA Extraction and Sequencing

RNA was extracted from samples harvested at 0 h, 4 h, 8 h, and 6 days, and the quality of RNA was checked as previously reported [2]. Sample collected at 0 h was used as the control. The cDNA library construction and sequencing were carried out by Metware Biotechnology Co., Ltd. (Wuhan, China). The clean reads obtained from all tested samples was assembled by Trinity software. Several commonly used databases, including NCBI, KEGG, NR, Tremble, Swiss-Prot, Pfam, KOG, and GO, were harnessed to annotate the unigene assembly. DESeq2 was utilized to screen DEGs by thresholds of |log_2_(fold change)| ≥ 1 and *p*-value < 0.01. To determine the function of DEGs, GO enrichment analysis was performed utilizing the top GO and KEGG pathways, and enrichment analysis was carried out by ClusterProfiler.

### 2.4. Quantitative Real-Time PCR

A Plant RNA Kit (Omega Biotek, Norcross, GA, USA) was used to extract total mRNA from samples, and then a UEIris II RT-PCR Kit (Novoprotein, Suzhou, China) was employed to synthesize cDNA by reverse transcription reaction. The NovoStart SYBR qPCR SuperMix Plus system (Novoprotein, Suzhou, China) was applied to analyze the expression level of generated cDNA. The primers used for qRT-PCR are listed in Table S1. All manipulations were carried out according to the manufacturer’s instructions. Using the endogenous actin gene as a standard, the expression levels of target genes was calculated by the 2^−ΔΔCt^ method [26].

### 2.5. Statistical Analysis

At least three biological replicates were employed for each parallel experiment, and data were presented as the mean ± standard deviation (SD). Statistical significance was considered at the *p*-value < 0.05 level using a *t*-test in Excel (Microsoft Office 2019).

## 3. Results and Discussion

### 3.1. Metabolomic Analysis

**Figure 1:**
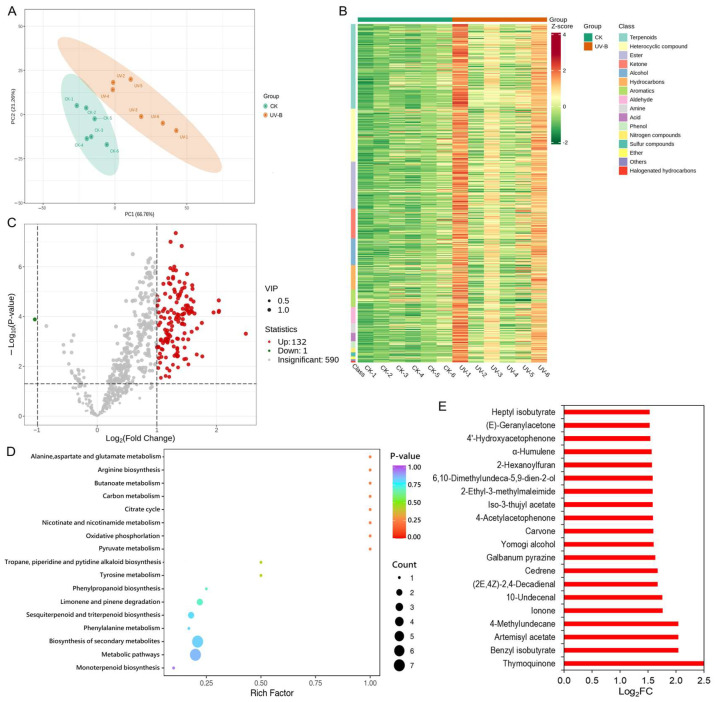
Overview of volatile metabolite changes in *A. argyi* leaves in response to UV-B radiation. (**A**) PCA of metabolites. (**B**) Cluster heatmap of all metabolite contents. The horizontal axis represents the sample name, and the vertical axis represents the metabolite information. Different colors are filled with different values obtained after standardizing the relative content (red represents high content, green represents low content). (**C**) Volcano plot of DEMs. (**D**) KEGG enrichment analysis of DEMs. (**E**) Top 20 upregulated DEMs.

To reveal the composition and variations of volatile compounds, a volatile metabolome analysis was performed on *A. argyi* leaf samples collected at 0 h and 6 days after UV-B radiation. In the principal component analysis (PCA) plot, the treatment samples (UV-B) and the control (CK) grouped separately (Figure 1A). A total of 733 metabolites were obtained in all samples. According to cluster analysis of the metabolite contents, volatile metabolites were classified into 16 categories (Figure 1B). Among the identified volatile metabolites, the proportion of terpenoids was the highest, followed by heterocyclic compounds, esters, ketones, and alcohols. It suggested that terpenoids might play a major role in response to UV-B radiation. Through statistical analysis, a total of 133 differentially expressed metabolites (DEMs) were identified, of which 132 metabolites were upregulated and one metabolite was downregulated (Figure 1C, Table S2). By KEGG enrichment analysis, the 133 DEMs were predominantly enriched in the following pathways: biosynthesis of secondary metabolites, metabolic pathways, sesquiterpenes and triterpenes biosynthesis, as well as limonene and pinene degradation (Figure 1D). The top 20 upregulated compounds included four terpenoids (ionone, cedrene, carvone, and humulene), four ketones (thymoquinone, 4-acetylacetophenone, 4′-hydroxyacetophenone, and (E)-geranylacetone), four esters (benzyl isobutyrate, artemisyl acetate, iso-3-thujyl acetate, and heptyl isobutyrate), three heterocyclic compounds (galbanum pyrazine, 2-ethyl-3-methylmaleimide, and 2-hexanoylfuran), two aldehydes (10-undecenal and (2E,4Z)-2,4-decadienal), two alcohols (yomogi alcohol and 6,10-dimethylundeca-5,9-dien-2-ol), and one hydrocarbon (4-methylundecane) (Figure 1E). Overall, terpenoids and heterocyclic compounds showed a more significant increase under UVB irradiation.

The generation of volatile metabolites is the result of the interaction between plants and biotic and abiotic factors during long-term evolution. Detailly, heterocyclic, phenolic, and flavonoid compounds can absorb UV radiation and provide photoprotection [27,28]. Terpenoids have antibacterial and anticancer activities and play an important role in the adaptation of plants to environmental stress [12,29]. A possible explanation for the accumulation of mono- and sesquiterpenes was that UV radiation directly affects the size and density of glandular trichomes, which are a special structure for storing and synthesizing aromatic compounds on the leaf [30]. As an important component of *Artemisia* essential oil, ketones possess antibacterial properties, which help plants to fight against bacterial infections [31]. Interestingly, most of the aforementioned compounds were methylated, which might be related to their important roles as signaling and structural molecules in plant defense and development [32].

### 3.2. Odorous Substances in Volatile Compounds

**Figure 2:**
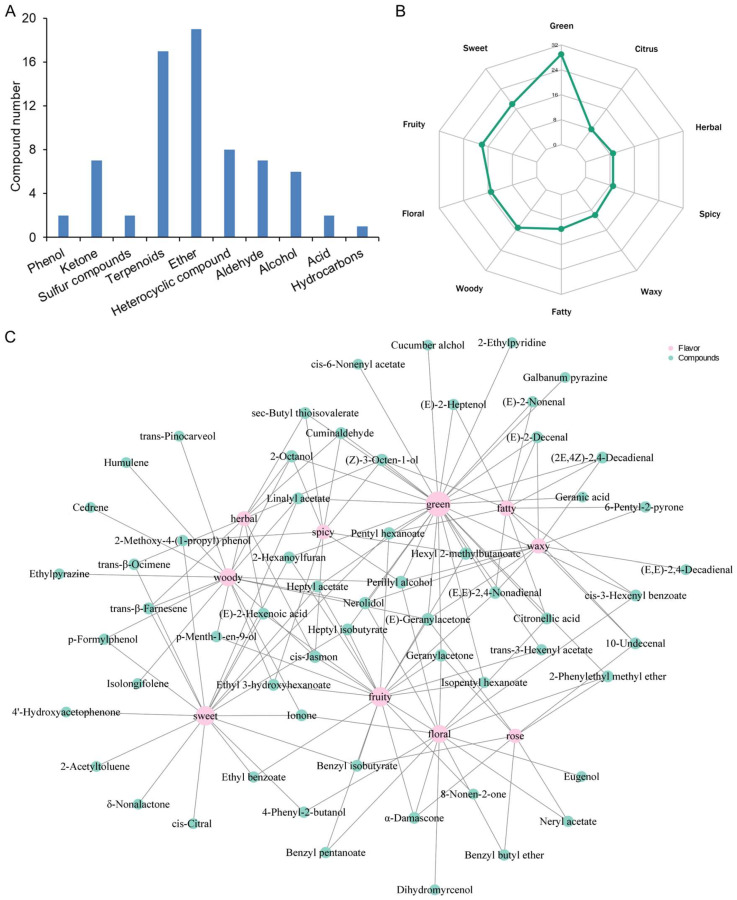
Odorous compounds analysis. (**A**) Category and number of DEMs with sensory flavor. (**B**) Radar chart of sensory flavor characteristics of differential volatile compounds. (**C**) Correlation network diagram between sensory flavor characteristics and DEMs.

The aroma components of plants can objectively reflect the flavor characteristics of different samples, and they are also important indicators for evaluating flavor and nutritional quality. Indeed, the unique fragrance of mugwort makes it a popular natural measure to repel mosquitoes and prevent epidemics. Therefore, it is necessary to analyze the volatile components and sensory odor of *A. argyi* in order to expand its application in the spice, food, and pharmaceutical industries. According to the sensory flavor analysis of 133 DEMs, a total of 73 DEMs were identified to have sensory flavors, with ether being the most abundant (19), accounting for 26.03% of the total (Figure 2A, Table S3). This was followed in descending order by terpenoids (17), heterocyclic compounds (8), ketone (7), aldehyde (7), alcohol (6), aromatics (2), acid (2), phenol (2), sulfur compound (2), and hydrocarbons (1). By further analysis of DEMs and annotated sensory flavor characteristics, the top 10 sensory flavors with the highest number of annotations were screened out among the 137 sensory flavors identified (Figure 2B). Green was the odor with the highest number of annotations, followed by sweet, fruity, floral, woody, and so on. Compounds with green odor include nine ethers (heptyl isobutyrate, pentyl hexanoate, sec-butyl thioisovalerate, trans-3-hexenyl acetate, cis-6-nonenol acetate, hexyl 2-methylbutanoate, heptyl acetate, isopentyl hexanoate, and cis-3-hexenol benzoate), five terpenoids (cuminaldehyde, perillyl alcohol, geranic acid, and nerolidol), four aldehydes ((2E,4Z)-2,4-decadienal, (E)-2-decenal, (E,E)-2,4-nonadienal, and (E)-2-nonenal), four alcohols (2-octanol, (E)-2-heptenol, 2,6-nonadienol, and (Z)-3-octen-1-ol), three heterocyclic compounds (2-hexanoylfuran, 2-ethylpyridine, and hazelnut pyrazine), two ketones (geranylacetone and (E)-geranylacetone), one aromatic (2-phenylethyl methyl ether), and one acid (citronellic acid). The relationship between sensory flavor characteristics and volatile compounds was further established (Figure 2C). Among the 73 volatile compounds with sensory flavor, except for five compounds with a single flavor, all other compounds possessed multiple sensory flavors.

Based on the above analysis, esters and terpenes were major contributors to the flavor, which was consistent with the findings in grape berries [22]. It is known that different esters have different aroma characteristics. Terpenoids with sensory flavor in *A. argyi* were predominantly monoterpenes and sesquiterpenes, which are not only efficient insect-repellent components of mugwort, but also play a key role in the formation of food flavors such as wine, fruits, and vegetables [21,22,29,33]. Some heterocyclic compounds, such as pyrazine compounds, mostly have strong nutty and baking aromas. In addition, terpenoids and heterocyclic compounds also possess various biological activities such as antioxidant, antibacterial, and stress resistance in plants [34,35,36]. Consequently, the key components of UV-B-induced flavor could be widely used in the cosmetic, pharmaceutical, and food industries. The above results indicated that UV-B could serve as a powerful tool to boost the sensory flavor of mugwort.

### 3.3. Illumina Sequencing and DEG Analysis

**Figure 3:**
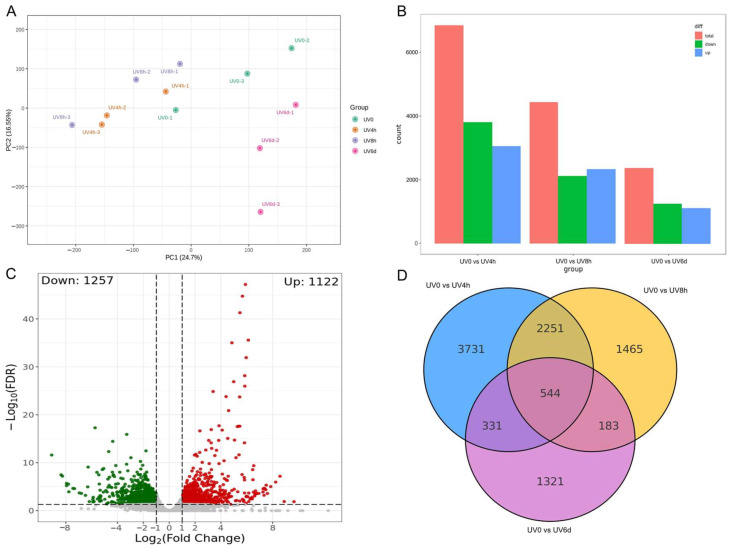
Overview of transcriptome analysis of *A. argyi* responsive to UV-B irradiation. (**A**) PCA analysis of samples taken at 0 h, 4 h, 8 h, and 6 days. (**B**) Changes in the total number of genes and DEGs. (**C**) Volcano map of DEGs from the pairwise comparison of UV0 vs. UV6d. (**D**) Venn graph for up- and downregulated DEGs from the pairwise comparisons of UV0 vs. UV4h, UV0 vs. UV8h, and UV0 vs. UV6d.

To reveal the potential molecular mechanisms underlying the changes in volatile compounds of *A. argyi* responding to UV-B radiation, transcriptome analysis was performed on samples treated with UV-B radiation for 0 h (control), 4 h, 8 h, and 6 days. The overall gene expression differences between each group of samples and the degree of variation between samples within the group can be preliminarily obtained by PCA. According to the results of PCA, the UV-treated samples and control could be discriminated, especially between the UV6d samples and the control (Figure 3A). Based on the value of log_2_(fold change) and *p*-value, different group comparisons (UV0 vs. UV4h, UV0 vs. UV8h, and UV0 vs. UV6d) were conducted to identify the differentially expressed genes (DEGs) and their expression level changes in *A. argyi* leaves after UV radiation. With the increasing UV irradiation time, the number of insignificant genes and upregulated and downregulated DEGs decreased to some extent, indicating that *A. argyi* gradually adapted this stress (Figure 3B). After 6 days of UV irradiation, the upregulated and downregulated DEGs reached 1122 and 1257, respectively (Figure 3C). The number of DEGs identified in this work also reflected the complexity of the *A. argyi* genome, which featured 62,000 genes through a recent whole-genome sequencing [2]. Venn plot analysis showed that 2795 DEGs were shared by two comparison groups, UV0 vs. UV4h and UV0 vs. UV8h (Figure 3D). The shared DEGs at the early stage of UV-B radiation might be related to the sensitivity of *A. argyi* to UV-B radiation. Moreover, 875 and 727 DEGs were obtained between the comparisons of UV0 vs. UV4h and UV0 vs. UV6d, as well as UV0 vs. UV8h and UV0 vs. UV6d, respectively. A total of 544 DEGs were shared by the above three pairwise comparisons, which might play a crucial role in *A. argyi*’s resistance and adaptation to UV-B radiation.

### 3.4. Metabolic Pathway Enrichment Analysis for DEGs

**Figure 4:**
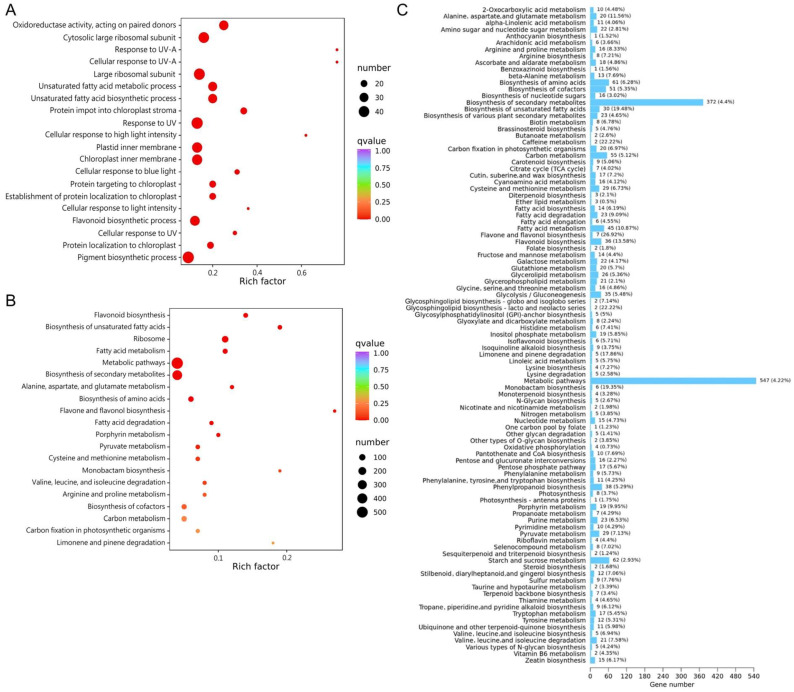
Enrichment analysis of metabolic pathways in comparison of UV0 vs. UV6d. (**A**) Top 20 enriched GO pathways of DEGs. (**B**) Top 20 enriched KEGG pathways of DEGs. The color and size of the solid circles represent the significant value of the enrichment factor and the number of transcripts involved in the specific pathway, respectively. (**C**) Classification of enriched metabolic pathways. The numbers in the figure represent the number of DEGs annotated to this pathway, and the parentheses indicate the ratio of DEGs annotated to this pathway to the number of background genes annotated to this pathway.

To elucidate the changes in volatile metabolites of *A. argyi* after 6 days of UV-B radiation, we analyzed the enriched biological pathways of DEGs using bioinformatic databases GO and KEGG [4]. GO enrichment analysis demonstrated that the DEGs after 4 h and 8 h of UV radiation were mainly enriched in “ribosomal subunit”, while the DEGs after 6 days of UV radiation were predominantly enriched for the terms “cytosolic large ribosomal subunit”, “large ribosomal subunit”, “response to UV”, “pigment biosynthetic process”, “plastid inner membrane”, “chloroplast inner membrane”, “flavonoid biosynthetic process”, and “unsaturated fatty acids metabolic process” (Figure 4A and Figure S1). The KEGG enrichment analysis revealed that “metabolic pathways” and “biosynthesis of secondary metabolites” were the most significantly enriched pathways after 4 h, 8 h, and 6 days of UV irradiation (Figure 4B and Figure S2). Compared with UV0 vs. UV4h, the profile of enriched KEGG pathways for UV0 vs. UV6d was more similar to UV0 vs. UV8h. The DEGs in the above three comparisons had common enrichment in “biosynthesis of secondary metabolites”, “flavone and flavonol biosynthesis”, “flavonoid biosynthesis”, and “fatty acid metabolism”. Most secondary metabolites, such as flavonoids, phenolic acids, and terpenes, are synthesized in defense against stresses [4,37]. The enrichment of DEGs supported this viewpoint. The discovery of significant changes in fatty acid metabolism induced by UV radiation was in line with previous work [38], indicating that the rapid variation in membrane lipids, especially unsaturated fatty acids, was crucial for cells to cope with multiple environmental stresses.

Further analysis showed that the most significant change in the proportion of DEGs to background genes annotated to this pathway was “flavone and flavonol biosynthesis”, followed by “caffeine metabolism”, “glycosphingolipid biosynthesis-lacto and neolacto series”, and “biosynthesis of unsaturated fatty acids” (Figure 4C). The most significant change in DEGs number was still “metabolic pathways”, followed by “biosynthesis of secondary metabolites”, “starch and sucrose metabolism”, and “biosynthesis of amino acids”. Secondary metabolites, especially phenolic compounds, were possibly the primary substances that plants produce in response to UV stress. Aromatic amino acids can be converted into phenylpropanoids, glucosinolates, and auxin, all of which participate in stress protection [39,40]. Glutamic acid and aspartic acid are precursors of other amino acids and involved in the tricarboxylic acid pathway (TCA) [41]. As such, the significant change in DEGs involved in “biosynthesis of amino acids” was linked to the subsequent accumulation of phenolics known for their antioxidant and UV-absorbing properties, as well as the re-programming of plant metabolism. The increased content of amino acids by UV-B radiation has also been reported in *Mentha spicata*, *Vitis vinifera*, and *Rhododendron chrysanthum* [23,28,42].

### 3.5. Integrated Analysis of Genes and Metabolites Involved in Volatile Compounds Biosynthesis

**Figure 5:**
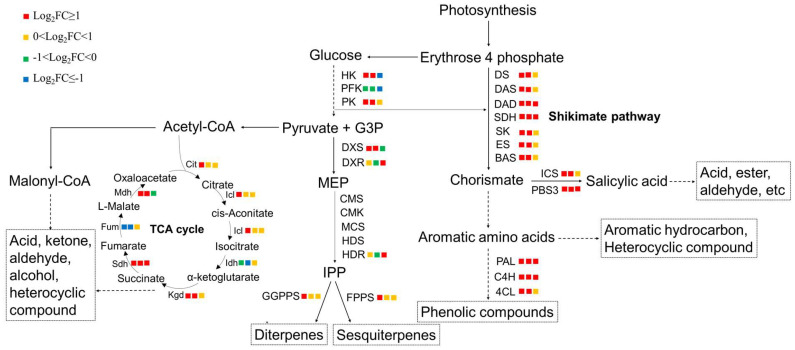
Metabolic analysis of volatile compounds in *A. argyi* leaves. HK, hexokinase; PFK, 6-phosphofructokinase; PK, pyruvate kinase; MEP, 2-C-methyl-D-erythrin-4-phosphate; IPP, isopentenyl pyrophosphate; DXS, 1-Deoxy-D-xylulose-5-phosphate synthase; DXR, 1-deoxy-d-xylulose-5-phosphate reductoisomerase; CMS, 2-C-methyl-D-erythritol 4-phosphate cytidine synthase; CMK, 2-C-Methyl-D-erythritol 4-phosphate cytidine kinase; MCS, 2-C-methyl-D-erythritol 2,4-cyclodiphosphate synthase; HDS, Hydroxymethylbutene-4-phosphate synthase; HDR, 1-Hydroxy-2-methyl-2 (E)-butenyl-4-diphosphate reductase; GGPPS, geranylgeranyl diphosphate synthases; FPPS, Farnesyl pyrophosphate synthase; Cit2, citrate synthase; Icl, isocitrate lyase; Idh, isocitrate dehydrogenase; Kgd, alpha-ketoglutarate dehydrogenase; Sdh, succinate dehydrogenase; Fum, fumarase; Mdh, malate dehydrogenase; DS, DAHP synthase; DAS, 3-dehydroquinic acid synthase; DAD, 3-dehydroquinic acid dehydratase; SDH, shikimate dehydrogenase; SK, shikimate kinase; ES, EPSP synthase; BAS, branched acid synthase; ICS, isochorismate synthase; PBS3, avrPphB susceptible 3; PAL, phenylalanine ammonia-lyase; C4H, cinnamate4-hydroxylase; 4CL, 4-coumarate-CoA ligase. The results were expressed as mean ± SD of triplicate measurements.

Through metabolome analysis, it was found that volatile compounds, particularly terpenoids and phenolic compounds, were raised in *A. argyi* leaves after exposure to UV radiation. Hence, the biosynthesis of terpenoids and phenolic compounds was analyzed in detail. As one of the main secondary metabolites, terpenoid is synthesized by the MEP pathway in plants. For the rate-limiting enzymes in the MEP pathway, 1-Deoxy-D-xylulose-5-phosphate synthase (DXS) was significantly upregulated at the transcriptional level during the initial stages of UV-B radiation, while the transcriptions of 1-deoxy-d-xylulose-5-phosphate reductoisomerase (DXR) and 1-Hydroxy-2-methyl-2 (E)-butenyl-4-diphosphate reductase (HDR) were significantly upregulated after 6 days of UV-B radiation (Figure 5). Isopentenyl pyrophosphate (IPP) is a key branch point for the synthesis of different terpenoid species. Geranyl diphosphate synthases (GPPS) and farnesyl pyrophosphate synthase (FPPS), responsible for the synthesis of monoterpenes and sesquiterpenes, were upregulated to varying degrees after UV radiation, matching the observed increase in the levels of monoterpenoids and sesquiterpenes. This was in accordance with reports that UV radiation boosted the synthesis of short-chain rather than long-chain terpenes [43]. Chorismate, a key precursor for the synthesis of salicylic acid and aromatic amino acids, is synthesized through the shikimate pathway. Genes encoding enzymes in the shikimate pathway were upregulated or significantly upregulated, suggesting that the precursor basis for the synthesis of aromatic compounds was strengthened by UV radiation. According to the latest reports, the isochorismate synthase (ICS) pathway is the predominant synthetic pathway for salicylic acid in plants [44,45]. The upregulated or/and significantly upregulated expression of key genes in the phenylpropanoid pathway (*PAL*, *C4H*, and *4CL*) and ICS pathway (*ICS* and *PBS3*) not only partially explained the reason why volatile components such as acids, ester, aldehydes, and phenolic compounds were enhanced under UV-B radiation, but also implied that the plant hormone salicylic acid played an important role in the adaptation of *A. argyi* to UV radiation.

Certainly, some volatile components such as acid, ketone, aldehyde, and alcohol might be generated by quadribasic acids from the TCA cycle, as UV-B radiation significantly upregulated genes encoding alpha-ketoglutarate dehydrogenase (KGD) and succinate dehydrogenase (SDH), which catalyze the production of succinic acid and fumaric acid, respectively. Other chemicals in cells, such as fatty acid derivatives and isoprenoids, were also utilized in the biosynthesis of volatile molecules [5,46]. In addition, methylation and ethyl modification occurred in most volatile molecules, which might be related to the increased gene expression of methyltransferases and acyltransferases (Figure S3). The above investigations reflected the complexity and diversity of volatile substance synthesis.

### 3.6. The Potential Regulation of Volatile Compounds by Transcription Factors

**Figure 6:**
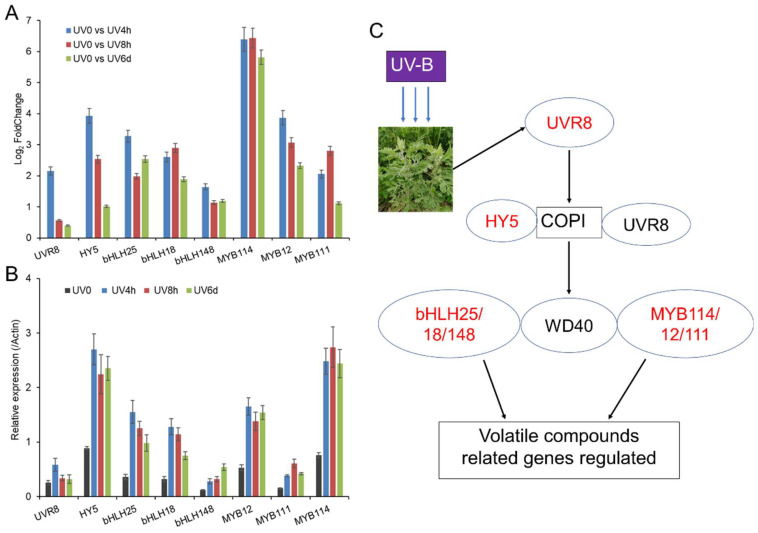
Transcriptional regulation of volatile compounds induced by UV-B. Gene expression of transcription factors analyzed by RNA-seq (**A**) and qPCR (**B**). Red characters indicate the upregulated metabolites. (**C**) A regulation model of volatile compounds-related genes. Red characters indicate the upregulated genes. Black characters indicate expression with insignificant differences. UVR8, UV Resistance Locus 8; COP1, Constitutively Photomorphogenetic 1; HY5, Elongated Hypocotyl 5.

Transcription regulation is one of the main ways of metabolic regulation. Plants are known to respond to UV-B radiation by the signaling transduction module of UV Resistance Locus 8 (UVR8), in conjunction with Constitutively Photomorphogenetic 1 (COP1) and Elongated Hypocotyl 5 (HY5) transcription factors [47,48]. To explore transcription regulators involved in the regulation of volatile compounds, we analyzed the expression of UVR8 signal transduction-related genes in response to UV-B radiation. The expression of *UVR8* was significantly upregulated after 4 h of UV-B radiation and upregulated after 4 h and 6 days of UV-B radiation (Figure 6A). Among all the transcription factors examined, only *HY5*, *bHLH25*, *bHLH18*, *bHLH148*, *MYB114*, *MYB12*, and *MYB111* were significantly upregulated at three time points. It indicated that the aforementioned transcription factors play a crucial role in the response of *A. argyi* to UV-B radiation. The results of qPCR confirmed the expression levels of *UVR8*, *HY5*, *bHLH25*, *bHLH18*, *bHLH148*, *MYB114*, *MYB12*, and *MYB111* (Figure 6B), showing the reliability of the RNA-seq data. Increased levels of *MYB* and *bHLH* can boost the expression of *PAL*, *C4H*, *PBS3*, and other genes, thereby enhancing the synthesis of secondary metabolites [49,50,51]. As a sensor of UV-B wavelength, UVR8 activity directly affects the expression of key genes in the terpenoid and flavonoid pathways [52]. Based on the above analysis, we proposed a regulation model of volatile compound in *A. argyi* responding to UV radiation (Figure 6C), in which transcription factors HY5, MYB, and bHLH family members might play a central role. Certainly, the response of plants to UV radiation can also be regulated by non-specific signaling pathways involving defense-induced hormones such as jasmonic acid and salicylic acid [4,10].

## 4. Conclusions

In the present study, the key volatile compounds and genes encoding-related enzymes and regulators of *A. argyi* in response to UV-B radiation were characterized by transcriptome and metabolomic analysis. A total of 133 DEMs and 544 DEGs were identified. DEMs were mainly composed of terpenoids, esters, heterocyclic compounds, alcohols, and ketones. Most DEGs were related to “metabolic pathways” and “biosynthesis of secondary metabolites”. Through integrated omics analysis, volatile compounds were mainly produced via the shikimate pathway and the MEP pathway, and a regulation model for the biosynthesis of volatile compounds in response to UV-B radiation was proposed, in which transcription factors HY5, bHLH25, bHLH18, bHLH148, MYB114, MYB12, and MYB111 were regarded as having a central role. This work provides valuable information on the composition and synthesis of volatiles in *A. argyi*, and facilitates the application of UV-B in the production of medicinal plants.

## Data Availability

The original contributions presented in this study are included in the article/Supplementary Materials. Further inquiries can be directed to the corresponding authors.

## Supplementary Materials

The following supporting information can be downloaded at: https://www.mdpi.com/article/10.3390/metabo14120700/s1, Figure S1: GO enrichment of DEGs in the comparisons of UV0 versus UV4h (A) and UV0 versus UV8h (B); Figure S2: KEGG enrichment of DEGs in the comparisons of UV0 versus UV4h (A) and UV0 versus UV8h (B); Figure S3: Transcriptional changes of methyltransferase (A) and acyl-CoA N-acetyltransferase (B) in leaves of *A. argyi* under UV-B irradiation. Table S1: Primers for qRT-PCR analysis; Table S2: DEMs in *A. argyi* leaves induced by UV-B radiation; Table S3: DEMs with sensory flavor in *A. argyi* leaves.
