# Supplementary material for: Enhanced Synthesis of Volatile Compounds by UV-B Irradiation in Artemisia argyi Leaves
Source: Metabolites. 2024 Dec 11;14(12):700. doi: 10.3390/metabo14120700 (PMC11678389; doi:10.3390/metabo14120700)
Supplement: Supplementary file 1 [file metabolites-14-00700-s001.zip › metabolites-3331515-supplementary figures.pdf]

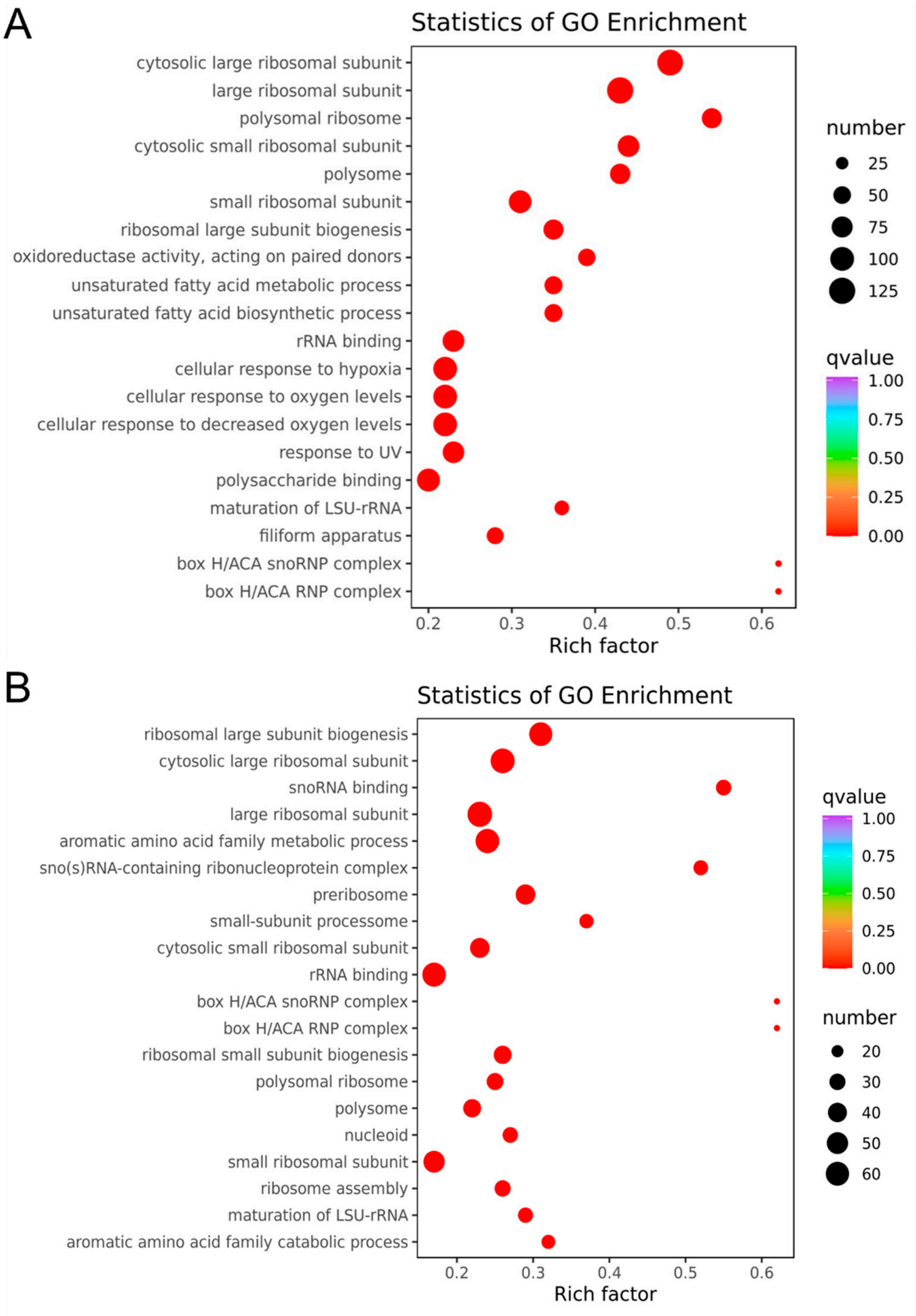

Figure S1 GO enrichment of DEGs in the comparisons of UV0 versus UV4h (A) and UV0 versus UV8h (B).

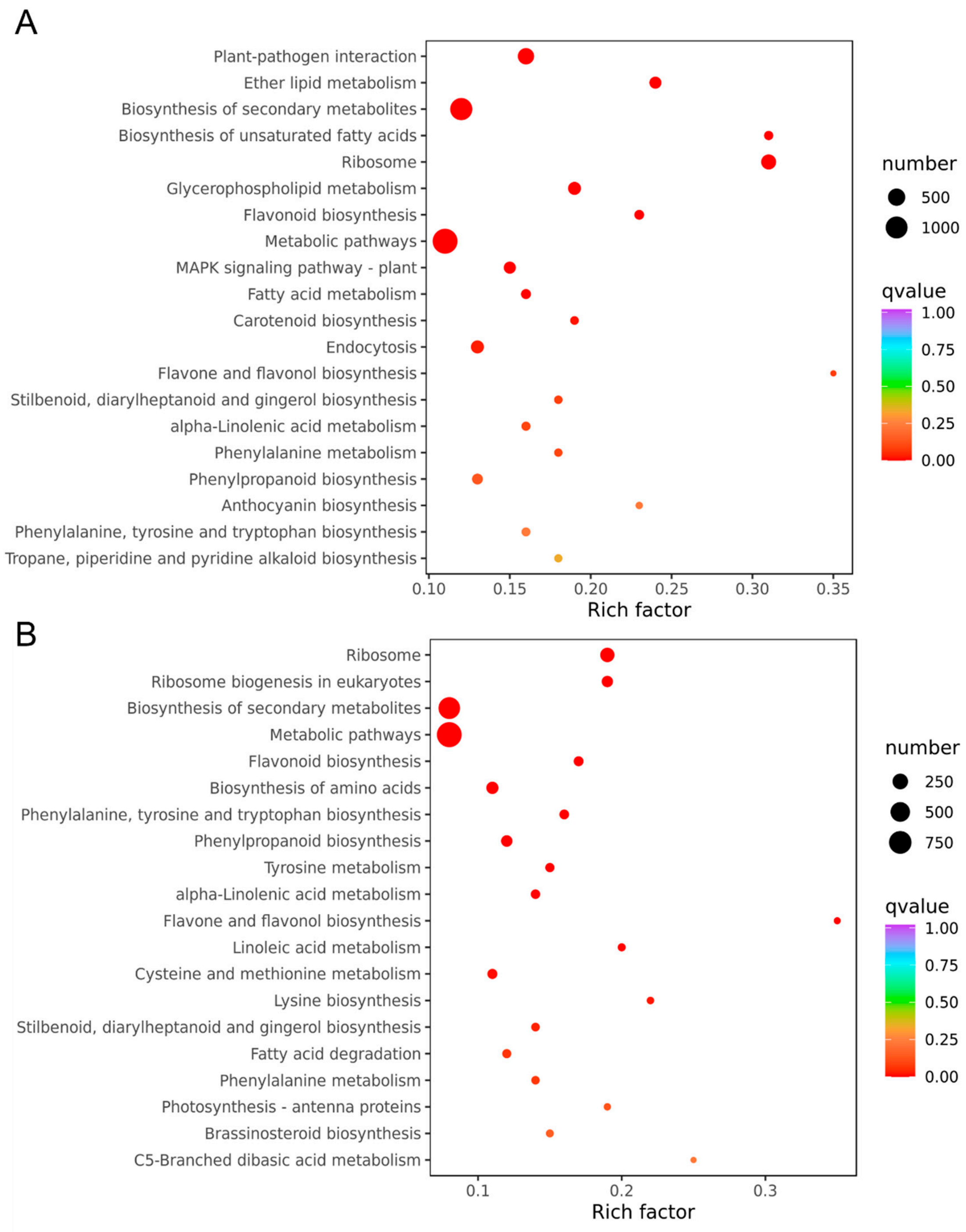

Figure S2 KEGG enrichment of DEGs in the comparisons of UV0 versus UV4h (A) and UV0 versus UV8h (B).

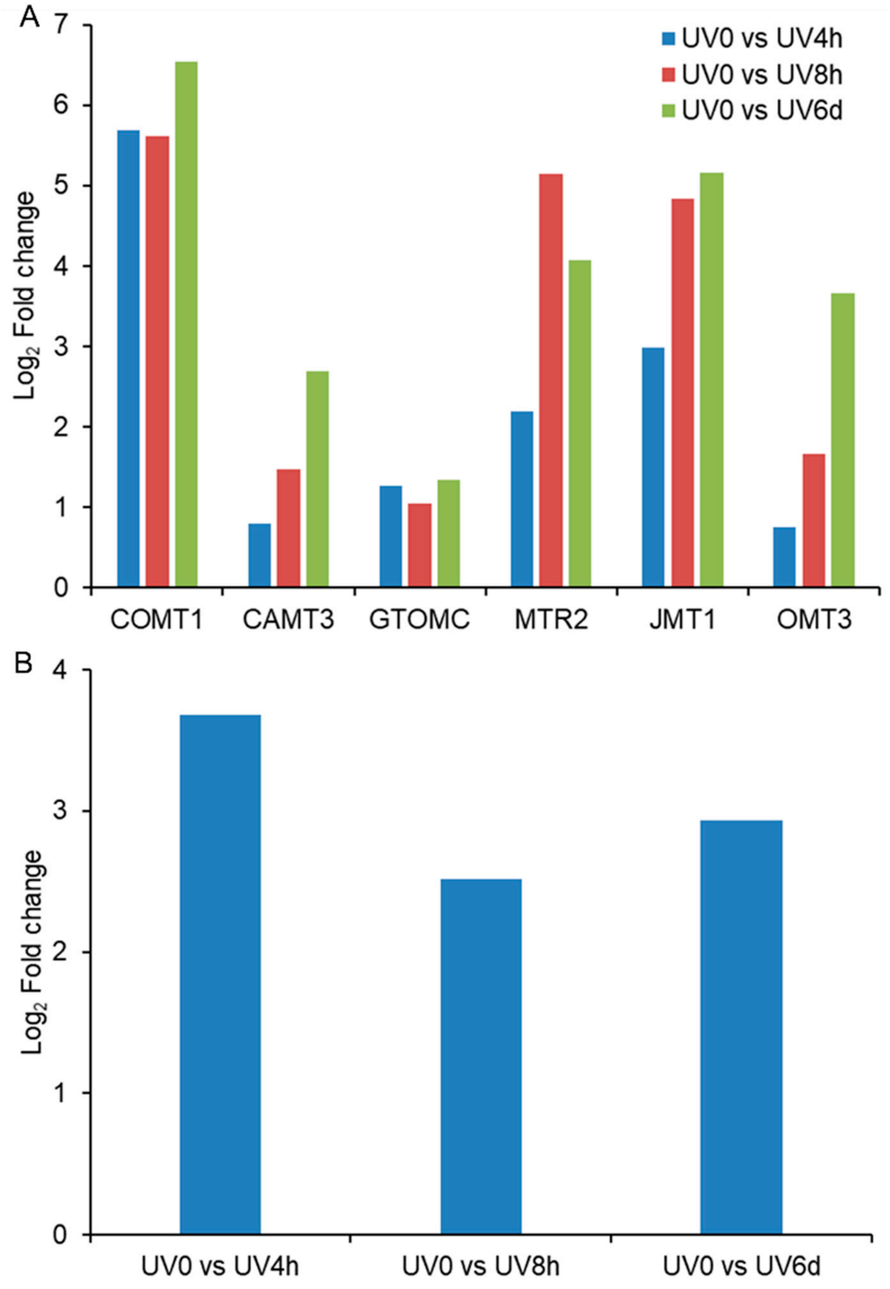

Figure S3 Transcriptional changes of methyltransferase (A) and acyl-CoA N-acetyltransferase (B) in leaves of *A. argyi* under UV-B irradiation. COMT1, caffeic acid 3-O-methyltransferase; CAMT3, S-adenosyl-L-methionine-dependent methyltransferases; GTOMC, tocopherol O-methyltransferase; MTR2, S-adenosyl-L-methionine-dependent methyltransferases; JMT1, jasmonic acid carboxyl methyltransferase 1; OMT3, O-methyltransferase 3.
